# ACCISS study rationale and design: activating collaborative cancer information service support for cervical cancer screening

**DOI:** 10.1186/1471-2458-9-444

**Published:** 2009-12-02

**Authors:** Ludmila Cofta-Woerpel, Veenu Randhawa, H Gene McFadden, Angela Fought, Emily Bullard, Bonnie Spring

**Affiliations:** 1 Department of Behavioral Science, UT M. D. Anderson Cancer Center and National Cancer Institute, Cancer Information Service, Unit 1330, P.O. Box 301439, Houston, TX, 77230, USA; 2 Maternal & Child Health Programs, Access Community Health Network, 222 N. Canal St., Chicago, IL 60606, USA; 3 Department of Preventive Medicine, Northwestern University, 680 N. Lakeshore Drive, Suite 1220, Chicago, IL, 60611, USA; 4 Department of Preventive Medicine, Northwestern University, 680 N. Lakeshore Drive, Suite 1400, Chicago, IL, 60611, USA; 5 National Cancer Institute, Cancer Information Service, University of Kansas Medical Center, 2100 W. 36th Ave, MS 3052, Kansas City, KS, 66160, USA

## Abstract

**Background:**

High-quality cancer information resources are available but underutilized by the public. Despite greater awareness of the National Cancer Institute's Cancer Information Service among low-income African Americans and Hispanics compared with Caucasians, actual Cancer Information Service usage is lower than expected, paralleling excess cancer-related morbidity and mortality for these subgroups. The proposed research examines how to connect the Cancer Information Service to low-income African-American and Hispanic women and their health care providers. The study will examine whether targeted physician mailing to women scheduled for colposcopy to follow up an abnormal Pap test can increase calls to the Cancer Information Service, enhance appropriate medical follow-up, and improve satisfaction with provider-patient communication.

**Methods/Design:**

The study will be conducted in two clinics in ethnically diverse low-income communities in Chicago. During the formative phase, patients and providers will provide input regarding materials planned for use in the experimental phase of the study. The experimental phase will use a two-group prospective randomized controlled trial design. African American and Hispanic women with an abnormal Pap test will be randomized to Usual Care (routine colposcopy reminder letter) or Intervention (reminder plus provider recommendation to call the Cancer Information Service and sample questions to ask). Primary outcomes will be: 1) calls to the Cancer Information Service; 2) timely medical follow-up, operationalized by whether the patient keeps her colposcopy appointment within six months of the abnormal Pap; and 3) patient satisfaction with provider-patient communication at follow-up.

**Discussion:**

The study examines the effectiveness of a feasible, sustainable, and culturally sensitive strategy to increase awareness and use of the Cancer Information Service among an underserved population. The goal of linking a public service (the Cancer Information Service) with real-life settings of practice (the clinics), and considering input from patients, providers, and Cancer Information Service staff, is to ensure that the intervention, if proven effective, can be incorporated into existing care systems and sustained. The approach to study design and planning is aimed at bridging the gap between research and practice/service.

**Trial Registration:**

NCT00873288

## Background

The burden of cancer falls most heavily on poor ethnic minorities - particularly African Americans and Hispanics [1]. Overcoming such disparities requires finding effective ways to increase the use and impact of scientifically accurate cancer information. Excellent, free resources, such as the National Cancer Institute's Cancer Information Service (CIS) exist and have potential to transmit needed information to underserved groups. However, despite greater awareness of the CIS by low-income African American and Hispanic adults, actual CIS usage is lower than expected, paralleling excess cancer-related morbidity and mortality for these subgroups [2]. The planned study will examine the efficacy of a targeted physician mailing designed to connect the CIS to low-income African American and Hispanic women who need cancer-related information due to a scheduled colposcopy to follow up an abnormal Pap test.

### Cancer Information Service

Established in 1975, the CIS is a health communication service designed to help meet the cancer information needs of the general public, health professionals, cancer patients, and their family members and friends [3]. Most users access the CIS via a telephone number (1-800-4-CANCER) and are connected to English- or Spanish-speaking information specialists. Information specialists are highly trained in both informational and the interpersonal aspects of cancer communication and are adept at addressing a wide range of cancer inquiries, including cancer prevention and control [2]. The CIS Partnership Program, dedicated to addressing cancer health disparities, works with partner organizations that have an established, trusted presence in the community. The CIS Research Program seeks to advance cancer related health communication science by designing, implementing, and disseminating results of innovative studies in a manner that bridges the gap between cancer research and service [4].

Awareness data from the 2003 National Health Information National Trends Survey suggest that the CIS is reaching the underserved. Awareness of the CIS was greater among minorities (51% of Hispanics, 45% of African Americans) than Whites (28%), contrasting with the opposite demographic awareness pattern for NCI, NIH, and ACS. Also, CIS awareness increased as income and education decreased and was greater among those without health insurance than those with it [2]. However, awareness data contrasted markedly with actual usage data showing that more Whites (83%) than African Americans (13.4%) or Hispanics (2%) called the service. These findings suggest barriers to use of the CIS by underserved minorities, despite good awareness.

The planned research stems from three premises. First, a strategic partnership between health care providers and the CIS can overcome call barriers and be advantageous to patients and providers [2,5]. Second, the CIS is ideally positioned to reach and meet the cancer information needs of the underserved, many of whom have telephone but no internet access. Third, the CIS can play an important role in overcoming communication barriers that contribute to cancer health disparities. Because the CIS enables continuous access to valid cancer-relevant information in the context of human interaction, implementing a strategic partnership between patients, CIS, and providers is consistent with the Institute of Medicine's call for care based on continuous healing relationships [6]. CIS offers a bridge between the underserved patients' preferred interpersonal source (the provider) and information that is available from family and friends in a low literacy community [7,8]. As such, CIS has the potential to positively impact multiple levels in an ecological model of public health communication [9,10].

### Effective public health communication

From an ecological perspective [9,10], disparities are grounded in a system of reciprocal influences at the intrapersonal, group, community, institutional, and population levels that impede optimal awareness, understanding, decision-making, and action about health. Effective public health communication interventions activate multiple channels to impact the different levels in the ecological framework [11]. Moreover, effective health communication initiatives make their outreach relevant to the intended audience [12]. The need to adopt an audience-centered perspective is especially strong when trying to connect with racial and ethnic minorities. Intrapersonal issues to be considered when making outreach to low-income African Americans and Hispanics include language and attitudinal barriers, low health literacy, lack of information, and misinformation about cancer screening, risk, and treatment [13-16].

Two widely used modes of audience-centric outreach are targeting and tailoring. ***Targeting***, which utilizes a market segmentation approach, creates messages to reach a specific population segment or group, often defined by shared demographics like ethnicity or language. Targeting of physician mailing in the proposed research will be performed on the basis of medical condition, clinic/ethnicity, and language. Of necessity, targeting addresses only surface, observable characteristics [17]. ***Tailoring***, in contrast, creates messages to reach a specific individual whose characteristics are determined by assessment. Because a skilled, non-time-constrained human can tailor messages much more effectively than a computer [18], CIS information specialists highly trained in interpersonal communication will be the tailoring agents for this study.

At an interpersonal level, the underserved express a strong cultural preference for receiving health information verbally [14,19] and primarily from health care providers [20-22]. In the current health care environment, those preferences increase informational dependence on overburdened providers who are accurately perceived by patients as lacking time for discussion [23]. Augmenting the communication challenges, providers spend less time with the underserved [24], at least partly because low literacy patients ask few questions of medical professionals even when language barriers are not an issue [19,25,26]. There is a tendency for both underserved patients and their providers to emerge from the medical encounter dissatisfied, and with patients feeling that their information needs have not been adequately addressed [27,28]. By virtue of the CIS's availability to the public, and the diverse and well-trained staff [29], information specialists potentially offer a vital resource to bridge this gap between underserved patients and their health care providers.

### Sustainability

Innovations at the institutional level occur most often when a recognized need and the means to resolve it emerge in tandem [30]. Such systems can be described as balancing "need-pull" with "technology-push". The time constraints of providers and unmet informational needs of low literacy patients represent a strong need that can be served by available CIS technology. A simple "push" in the planned intervention is the provider letter that alerts the patient to a medical risk and an information need. Explaining that the CIS call is to be discussed at the upcoming medical appointment frames the CIS in a culturally acceptable normative context as a provider-extender rather than an outsider. Patients who are activated to discuss CIS calls at the medical visit give a reciprocal "push" that helps educate providers. By suggesting questions to ask of the CIS, the intervention prompts patients to learn about information gathering behavior, creating communication skills that may generalize to the medical encounter. The intervention is sustainable as a part of institutionalized office practice [31,32].

To maximize public health impact, the CIS needs to reach large new populations of underserved individuals whose cancer information needs are presently unmet [33]. The population at risk for cancer represents a good target because it is larger than the population of those already diagnosed with cancer. Thus, the planned intervention has potential to be sustained and increase CIS impact among the underserved.

### Cervical Cancer Disparities

African American women have both increased incidence and the highest rate of mortality from cervical cancer, showing mortality rates more than twice that of non-Hispanic Whites [34]. Survival is also lower among Hispanics, largely because of more advanced cancer at the time of diagnosis [1]. As many as 50% of medically underserved women in the National Breast and Cervical Cancer Early Detection Program failed to receive appropriate follow-up for an abnormal Pap smear, with the likelihood of no follow-up being greatest for African American women [35]. Another recent study of the urban poor found an inadequate follow-up rate of 38%, despite opportunities for follow-up since 86% of women continued to receive care in the same place [36].

Misinformation, lack of information, fear, and embarrassment are all barriers to receiving adequate follow-up care. Predictors of good follow-up are remembering receiving a letter from the provider, knowing the Pap result, being able to report the result correctly, understanding the purpose of colposcopy, having low fear of pain, and feeling confident that the provider will understand the patient's needs [37,38]. Consistent with the health belief model [39], therefore, accurately perceiving one's susceptibility to cervical cancer, feeling confident about being able to take protective action, and perceiving prognostic benefits (versus fatalism) of follow-up for early detection are associated with better follow-up. Telephone outreach addressing these domains has been demonstrated to be effective in increasing follow-up adherence to colposcopy by low-income, inner-city women [40]. An innovation in the proposed research is that telephone support will be provided by CIS information specialists.

### Study Aims

Using formative and experimental research methodologies, the planned research will investigate how to connect the CIS to low-income urban African-American and Hispanic women with abnormal PAP tests. Specific aims are to examine whether a targeted physician mailing can increase calls to the CIS, enhance appropriate medical follow-up, and improve satisfaction with provider-patient communication at the follow-up medical visit.

## Methods/Design

### Study sites and phases

Study procedures were reviewed and approved by Northwestern University's Office for the Protection of Research Subjects Institutional Review Board. The research will be conducted in two large community clinics that serve poor, ethnically diverse communities in Chicago. At the Erie Family Health Center (EFHC) on Chicago's West side, 82% of clients live at or below the federal poverty level; 57% have no health insurance; 90% are Hispanic or Latino. At Prentice Ambulatory Clinic (PAC) on Northwestern's Chicago campus, outpatient services are provided to a population that is 90% Medicaid/Medicare and 85% minority (60% African American, 30% Hispanic).

The initial, formative phase of the research will solicit input from patients and providers regarding all materials planned for use in the experimental phase of the study (e.g. questionnaires, consent procedures, and intervention materials). The experimental phase will follow comparing the intervention and control conditions.

### Formative phase

The formative research phase will acquaint the project team with the clinics. Next, focus groups with patient and providers will be conducted in each of these clinics.

#### Participants

Participants for the formative phase will be selected to be representative of the population at each clinic. Inclusion criteria for patients will be women over the age of 18 years who have had a pap test in these two clinics and who agree to participate. Inclusion criteria for providers will be any provider who administers pap tests and conducts colposcopy testing for women at the two clinics and are available to participate. Patients and providers will be consented prior to conducting the focus groups.

#### Focus groups

The project staff will conduct 4 focus groups of 5 participants each: 5 patients from EFHC, 5 providers from EFHC, 5 patients from PAC, and 5 providers from PAC. The patient focus group at EFHC will be co-facilitated by staff that is fluent in Spanish. Topics to be discussed in the focus groups are: a) perceived information support needs and barriers to following up abnormal Pap tests (e.g., fears, embarrassment, misinformation, kinds of information and messages desired, kinds of messages deemed unhelpful), b) feedback on the letters and assessment instruments planned for the randomized controlled trial (RCT) (e.g., reactions to calling the CIS service, reactions to inclusion of the word 'cancer' in the name of a service offering preventive care), c) familiarity with and views about the CIS, d) barriers and facilitators to telephoning the CIS for information about cancer prevention and screening. Focus groups will be audio taped to ensure the discussions are completely and accurately recorded.

#### Analysis

Focus group data will be transcribed verbatim by project staff. Staff fluent in Spanish will translate the Spanish focus groups in English and then transcribe them. Transcribed data from focus groups will be analyzed and interpreted via thematic analysis [41]. Three raters will read the transcribed materials thoroughly to identify themes for each topic. Codes for these themes will be developed and the responses will be tabulated using the coding scheme. Lessons learned will be discussed and will help guide tailoring the letter and assessment instrument for the RCT.

### Experimental phase

#### Study Design

The experimental phase of the study will be conducted using a two-group prospective RCT design comparing the effects of two provider mailing conditions: control (Usual Care) versus intervention (CIS + Questions). The RCT design will be 2 × 2 factorial with Clinic (EFHC versus PAC) and Letter (Usual Care versus Intervention) as between subjects factors.

#### Participants

This study aims to recruit 500 women with an abnormal annual Pap test and follow-up scheduling for colposcopy at EFHC or PAC. Based on the current patient flow in the two clinics, we expect to randomize approximately 100 women from EFHC and 400 from PAC. Entry criteria will include women of age 18 or older who speak either English or Spanish and who have received an annual Pap test that warrants scheduling for colposcopy at EFHC or PAC. Women scheduled for a repeated Pap test or repeated colposcopy as follow up to a previous abnormality will not be enrolled because they may already have completed their initial information seeking process.

#### Randomization

Patients will be randomized to two conditions that involve different letters from the provider. *Usual Care *participants will receive a generic letter reminding them of their follow-up appointment. *Intervention *participants will receive a targeted letter reminding them of the follow-up appointment, asking them or someone they designate to call CIS, and suggesting some questions to ask.

The patient will be the unit of randomization and the project statistician will prepare and manage the randomization scheme. Separate randomizations will be performed for EFHC and for PAC. Usual Care and Intervention letters will be placed in envelopes. At the time randomization envelopes are selected, group allocation will be concealed from study staff and clinic staff so that there is no selection bias in the randomization process.

#### Intervention

As soon as the patient has received notification of the lab Pap test result and has been scheduled for a colposcopy appointment, the appropriate control or intervention letter from the provider will be sent, along with a letter informing the patient about the study. The Usual Care letter from the provider will confirm the abnormal Pap and remind the patient of the scheduled follow-up appointment for colposcopy. The Intervention (CIS + Questions) letter from the provider will convey the same appointment information as the Usual Care letter. It will, in addition, ask the patient or a designate to telephone 1-800-4-CANCER and it will suggest some questions to ask about abnormal Pap tests and colposcopy. The suggested questions are: "What does an abnormal Pap test mean?", "What is colposcopy and what can I expect during colposcopy?", and "How will it benefit me to take the test?"

Consistent with the Institute of Medicine's [42] recommendation, the letters will be appropriate for reading skills at or below an eighth-grade level. The Lexile, a measure of reading difficulty, will be used to gauge reading level [43,44].

Letters will be written in the patient's primary language, Spanish or English. They will be mailed on PAC or EFHC letterhead with a signature per the patient's provider. Text will be typed double-spaced in large font, filling less than a page. Like all materials planned for use in the RCT, the proposed letters will be reviewed with community members and providers during the initial formative phase of the study. The final content of the Usual Care and Intervention letters will be based on input from patients and providers.

#### Outcomes and measures

##### Primary outcomes

There are three primary study outcomes 1) telephone calls to CIS (call volume); 2) timely completion of medical follow-up (within 6 months); 3) patient satisfaction with doctor-patient communication at medical follow-up.

##### Telephone calls to the CIS

The CIS will tabulate calls originating from the study. When CIS information specialists answer the telephone, they log call and caller information into a standard Electronic Call Record Form (ECRF). With the caller's consent, three customer service questions are asked of all callers:

1. Have you used our service before?

2. How did you find our number to call?

3. What is your home ZIP code?

The ECRF response field for Question #2, "How Found Out" (HFO) has many codes, e.g., 300 - TV, 400 - radio, 801- ACS etc., including blank codes that can be assigned to a specific promotion or research study. Four How Found Out response codes will be allocated to identify the conditions in this study: Code 1: PAC, Usual Care; Code 2: PAC, Intervention; Code 3: EFHC, Usual Care; Code 4: EFHC, Intervention.

Information specialists will follow a series of steps (see: ECRF programming below) to determine which, if any, of the four codes should be assigned to a call.

Operationalizing call tracking in this way produces de-identified data. Consequently, it will not be possible to link ECRF-coded call data to the patient's other individual level outcome data. To accomplish such linking, self-reported data on calls to CIS also will be collected from identified participants during the exit interview. This will allow exploratory examination of whether calling the CIS appears to mediate more timely follow-up and improved provider-patient interaction.

##### Timely medical follow-up

Scheduling of colposcopy appointments will be tracked electronically and by exit interview. Timely medical follow-up will be operationalized by whether the patient keeps an appointment for colposcopy within six months of the initial abnormal Pap. Colposcopy is the primary disposition for abnormal Pap tests at EFHS and PAC. Clinic staff endeavor to schedule all patients within three months of the abnormal Pap. Colposcopy is performed on-site at both EFHS and PAC, in order to maximize continuity and follow-up. The procedure may be performed by the patient's primary care physician or by one of several doctors at each clinic who specialize in colposcopy.

##### Patient satisfaction with provider-patient communication

Satisfaction with provider-patient communication will be assessed by the *Doctors Who Communicate Well *scale from the 2.0 Adult Core Survey of the Agency for Healthcare Research and Quality's *Consumer Assessment of Health Plans Study (CAHPS*^®^) [45-47]. The CAHPS scale consists of four items asking the degree to which the provider: listens carefully, explains things understandably, shows respect, and spends enough time with the patient. Each item has 5 response options: never, sometimes, usually, always, I didn't see the provider. The scale's developers recommend collapsing responses to a 3-category distribution by combining "never" and "sometimes" into a single category [48] yielding a 1-3 range per item, where higher numbers indicate better care. Validated in both English and Spanish [45-47], the *Doctors Who Communicate Well *scale shows excellent internal consistency (Chronbach's alpha = .86) and predicts the rated quality of health care [45]. Patients will complete the CAHPS immediately after the follow-up visit for colposcopy.

##### Ancillary outcomes

To support exploratory analyses, patients and providers will be asked several additional questions at the medical follow-up visit.

##### Patient exit interview

After completing the CAHPS, patients will be asked: 1) whether they telephoned the CIS, and if so, how many times; 2) whether any others telephoned the CIS on their behalf, and, if so whom; 3) their satisfaction with the CIS call experience; 4) other ways in which they sought information about screening; 5) whether the doctor who performed the colposcopy was their regular physician; 6) whether family members accompanied them to the visit.

##### Provider exit interview

Because there is, to our knowledge, no existing short scale to measure a patient's skill in communicating with providers, we developed a checklist of patient communication behaviors modeled after the SEGUE Framework [49]. The SEGUE checklist of provider communication tasks is the most widely used tool for physician communication training in North America. The six items to be rated Yes/No are:

No Yes

A) Showed some understanding of Pap/colposcopy

B) Paid attention to what you said

C) Asked 1+ questions

D) Talked about concerns

E) Expressed interest in caring for her health

F) Used time efficiently

*Language barrier and no interpreter

##### Maintenance

Six months after project staff has discontinued the initiative to randomize patients, clinic staff will be interviewed to determine whether either clinic has continued to use the tailored mailing.

### Procedure and data collection

#### Protocol sequence

Clinic staff at both clinics will monitor Pap test results to identify patients eligible for this study. Based on patient flow data, we expect to be able to randomize approximately 100 women from EFHC and 400 women from PAC. Participants will be randomized to two conditions in which they will receive one of two letters previously described: Usual Care participants will receive the general reminder letter about their colposcopy appointment and Intervention participants will receive the reminder letter, plus a request to call the Cancer Information Service (1-800-4-CANCER) and suggested questions to ask.

An in-person informed consent process will be undertaken at the colposcopy appointment. Upon arrival for the appointment, clinic staff will remind patients about the letter, inform them about the study, and ask if they are interested in further information. Those interested in further information will be approached by research staff and given a detailed description of the study, emphasizing that participation is completely voluntary. Participants who agree to participate and provide written consent will be enrolled in the study and will receive an exit interview.

#### CIS data collection

Once CIS's usual service is complete, the information specialist will ask the customer service questions in the order listed previously. ECRF will be programmed in such a way that a zip code which falls within the study area will trigger an electronic prompt for the information specialist to ask a series of probing questions. The goal of the probing questions will be to determine if the caller is a part of the study population and if so, from which clinic and which study group (Intervention versus Usual Care). The HFO code will be re-coded to a study code accordingly.

The first probing question is, "Have you or someone you know recently had an appointment at Prentice Ambulatory Clinic or Erie Family Health Center in Chicago? If yes, which clinic?" If the caller responds "no," then a non-study HFO code is used and no more probing questions are asked. If the caller responds either "yes, PAC" or "yes, Erie" the second probing question is asked.

The second probing question is, "Did you or someone you know recently receive a call or letter about having a colposcopy for a positive pap test?" If the answer is "no," then a non-study HFO code is used. If the caller answers, "yes," then the third probing question is asked.

The third probing question is, "Did your letter or telephone call ask you to call 1-800-4-CANCER?" If the answer is "no," then the call is coded as either PAC Usual Care or EFHS Usual Care, depending on the caller's answer to the first probing question. If the caller answers "yes," then the call is coded as either PAC Intervention or EFHS Intervention, depending on the caller's answer in the first probing question.

### Statistical analysis plan

#### Quality control and data management

Data management and quality control will be the joint responsibilities of the study coordinator and the laboratory data manager, utilizing the resources of the Biostatistics Core Facility of the Robert H. Lurie Comprehensive Cancer Center. Study databases in Excel and SPSS will be developed to house all study data. Databases, stored on the local PCs of the data manager and study coordinator, will be encrypted and password protected. Entered data will be monitored and questionable values will be checked and corrected as appropriate. Randomization balance will be evaluated quarterly to assure equal sample sizes in the study groups.

#### Statistical analysis

Based on information provided by site providers, we estimate a final N of 500 (250 intervention, 250 control) consented for follow-up to be reasonable and achievable within the one-year recruitment window. The study has four specific aims:

**Aim 1 **is to conduct focus groups about the CIS, and the targeted study letter with 10 patients and 10 providers from two large clinics that serve low-income women in Chicago. Transcripts of the focus groups will be analyzed qualitatively for content to determine the totality of ideas that emerge from the discussions. All data will be summarized descriptively. In particular, information will be compiled on familiarity with and views about the CIS, barriers and facilitators to telephoning the CIS for information about cancer prevention and screening and perceived information support needs for following up abnormal Pap tests.

**Aim 2 **is to randomize and enroll all women between ages 18-60 who receive an abnormal annual Pap test with follow-up scheduling for colposcopy at two clinics for underserved women. Randomization to Usual Care or Intervention will be conducted using the previously described procedure.

**Aim 3 **is to compare the number of telephone calls made to the CIS by patients randomized to Intervention versus Usual Care in order to test the hypothesis that a targeted physician mailing that asks patients to call the CIS and suggests questions will increase CIS call volume compared to Usual Care control. The outcome measure will be whether or not a study participant calls the CIS. Proportions will be compared between study groups using the Cochran-Mantel-Haenszel chi-square test [50]. With 250 persons per group, there is 80% power to detect a difference of a 5% call rate in the control group versus a 12% call rate in the intervention group. There is greater than 99% power to detect the anticipated difference of 5% in the control group versus 40% in the intervention group.

**Aim 4 **is to compare the Intervention and Usual Care groups' medical follow-up in the 6 months after abnormal Pap test notification and exit interview data at Pap test follow-up. The two outcome measures will be the dichotomous variable of timely follow-up completion (yes, no) as well as the CAHPS satisfaction questionnaire. The proportion of participants completing follow-up within 6 months will be compared between study groups using the Cochran-Mantel-Haenszel chi-square test. It is anticipated that 450 participants will consent to participation. With 225 persons per group, there is 80% power to detect a difference between a 55% appropriate follow-up rate in the control group versus a 68% rate in the intervention group. There is 99% power to detect the anticipated difference of 55% in the control group versus 75% in the intervention group.

To compare patient satisfaction between the two groups, the four-item CAHPS scale will be used where each item is coded 1 (never, sometimes), 2 (usually) or 3 (always). The sum of these four items will have a range of 4-12. If this range represents 4 standard deviation units, then a conservatively large estimate of the standard deviation is 2 units on the CAHPS 4-item scale. Anticipated sample size for the analysis of satisfaction data (given expected follow-up rates of 55% control and 75% intervention) is 293 (control N = 124, intervention N = 169). These sample sizes have 80% power to detect an effect size of 0.34, where effect size is defined as the mean difference between intervention and control, divided by the standard deviation. With an estimated standard deviation of 2, this effect size translates into a mean difference of 0.68 (.34 × 2). With these assumptions for the sample size, the study is powered to detect a consistent difference between the groups of one response category for one item. Before the satisfaction data are analyzed, participant characteristics will be compared between the control and intervention subsamples to determine any selection bias that may have occurred due to differential follow-up between the groups. Satisfaction data will then be analyzed using a two-factor analysis of variance, with clinic (EFHC versus PAC) as one between-subjects factor and study condition (Usual Care versus Intervention) as the other. The interaction term will determine whether the intervention effect is different between the two clinics. Any demographic characteristics found to be different between the groups will be included in this analysis as covariates. Other potential covariates are whether the physician is patient's usual provider, language barrier, availability of needed translation, and whether family members accompany the patient to the appointment. The intervention by ethnicity (Hispanic vs. African American) interaction will also be tested: prior studies neither support nor negate such interaction. For all power calculations, a two-tailed test and a Type I error rate of 5% is assumed.

## Discussion

The present study will develop and test the efficacy for enhancing calls to the CIS, timely colposcopy, and satisfaction with provider-patient communication of a targeted physician mailing. The mailing is designed to connect the CIS to low-income African-American and Hispanic women who need information about abnormal Pap tests and colposcopy. The study has the potential to identify a low-cost, feasible, effective, sustainable, and culturally sensitive strategy to increase awareness of the CIS and use of its free cancer information services among an underserved population. In addition to rigorous efficacy testing of the intervention during the experimental phase of the research, the study will examine other aspects of feasibility such as acceptability, and demand [51]. The formative phase of the research evaluates the acceptability of the intervention via focus groups that ask patients and providers about the design and perceived usefulness of the proposed targeted physician mailing. Calls to the CIS from patients participating in the study, a primary outcome in efficacy testing, also can be interpreted as a measure of demand for the intervention among the target population. In this study, demand will be assessed both by self-reported calls and calls recorded by the CIS.

By testing a feasible intervention in real-life settings of practice (the clinics) and public service (the CIS), and by evaluating different aspects of feasibility, the ACCISS study aims to bridge a gap between research and practice. So few trials on the efficacy of cancer prevention and control interventions have been tested under real-world conditions so as to suggest to some that randomized controlled trials have low applicability to public health settings [52-54]. The ACCISS study proceeds from the premise that high quality randomized controlled trials can be implemented quite feasibly in a real world setting. Indeed, we suggest that it would be inequitable to test interventions intended for underserved populations using research designs less internally valid than those used to test interventions intended for majority populations. Patients, providers, and CIS staff all will participate in the development of the intervention to ensure its acceptability, culturally sensitivity, and sustainability. Making dissemination potential and practice integration a priority from the inception of the research, CIS and clinic representatives were involved in the development of the proposal for funding. Finally, the inclusion of adherence and patient satisfaction as primary outcomes is congruent with recommendations to include multiple, clinically relevant outcomes in practical clinical trials [52,55]. The research follows these foundational principles so that, if proven effective, the ACCISS intervention can be sustained and referrals to the CIS can be incorporated into clinical practice. CIS is well positioned to enhance and supplement cancer-related information provided in primary care settings in a manner that benefits both patients and health care providers. CIS's status as part of the National Cancer Institute, addresses potential provider concerns regarding quality of the information provided by a third party. By offering information in one-on-one interactions with specialists well- trained in the interpersonal as well as informational aspects of cancer communication, CIS has potential to invoke trust among underserved patients who as a rule prefer to receive health information verbally [14,19].

CIS is also favorably positioned to partner in the complex endeavor of research. Although it is first and foremost a service organization, CIS has its own research program complete with a research agenda dedicated to bridging the gap between research and service [4]. CIS staff is trained to conduct research studies embedded in the service environment. From the perspective of CIS, this study is a model of collaboration. The research engages CIS with academic researchers and public health clinics in a mutually beneficial collaboration that strives to produce health benefits for the underserved.

While the study has considerable strengths, it also has limitations. First, in the clinics and within the CIS, practice/service takes priority over research. Therefore, extra effort will be required to integrate research into daily practice and service activities in a manner that ensures correct implementation of data collection processes. Second, a primary outcome, calls to the CIS, will be measured via patient self-report as well as via calls recorded by CIS as coming from the study. Because CIS calls are anonymous, call information cannot be used to verify self-report or vice versa.

These limitations notwithstanding, the present study has the potential to connect the CIS to low-income patients who need cancer screening information. By doing so, the study may contribute to overcoming communication barriers that underlie cancer health disparities.

## Competing interests

The authors declare that they have no competing interests.

## Authors' contributions

All authors contributed to the study design and development of the trial protocol. BS is the principal investigator of the study and LCW is a co-investigator and liaison between the CIS and the study team. VR is the study coordinator and EB is responsible for training CIS information specialists in coding calls from the study. HGM is the data manager and performed data quality control. AF prepared the randomization and reviewed the statistical analysis plan. All authors contributed to writing and revising the paper. All authors read and approved the final version of the manuscript.

## Pre-publication history

The pre-publication history for this paper can be accessed here:

http://www.biomedcentral.com/1471-2458/9/444/prepub
